# Crystal structure of a family 6 cellobiohydrolase from the basidiomycete *Phanerochaete chrysosporium*

**DOI:** 10.1107/S2053230X17008093

**Published:** 2017-06-17

**Authors:** Mikako Tachioka, Akihiko Nakamura, Takuya Ishida, Kiyohiko Igarashi, Masahiro Samejima

**Affiliations:** a Department of Biomaterial Sciences, Graduate School of Agricultural and Life Sciences, The University of Tokyo, 1-1-1 Yayoi, Bunkyo-ku, Tokyo 113-8657, Japan; b VTT Technical Research Centre of Finland, PO Box 1000, Tietotie 2, Espoo FI-02044 VTT, Finland

**Keywords:** cellulases, *Phanerochaete chrysosporium*, cellobiohydrolase, biomass utilization, carbohydrate-active enzymes

## Abstract

Cellobiohydrolases belonging to glycoside hydrolase family 6 (CBH II, Cel6A) play key roles in the hydrolysis of crystalline cellulose. CBH II from the white-rot fungus *Phanerochaete chrysosporium* (*Pc*Cel6A) consists of a catalytic domain (CD) and a carbohydrate-binding module connected by a linker peptide, like other known fungal cellobiohydrolases. In the present study, the CD of *Pc*Cel6A was crystallized without ligands, and *p*-nitrophenyl β-d-cellotrioside (*p*NPG3) was soaked into the crystals. The determined structures of the ligand-free and *p*NPG3-soaked crystals revealed that binding of cellobiose at substrate subsites +1 and +2 induces a conformational change of the N-terminal and C-terminal loops, switching the tunnel-shaped active site from the open to the closed form.

## Abstract 2

The crystal structure of the catalytic domain of a glycoside hydrolase family 6 cellobiohydrolase from the basidiomycete *P. chrysosporium* was solved in apo and cellobiose-liganded forms at 1.2 and 2.1 Å resolution, respectively.

## 1. Introduction   

Cellulose is the most abundant biopolymer on Earth, and is a potential alternative resource to fossil-based fuels and chemicals. It consists of β-1,4-linked d-glucose units, and is mainly found in plant cell walls in highly crystalline forms, which provide mechanical strength and resistance to microbial and chemical breakdown. Cellulose-degrading microorganisms have evolved to produce an array of extracellular enzymes that convert cellulose into soluble oligosaccharides. Cellobiohydrolases (EC 3.2.1.91), which hydrolyze cellulose to cellobiose (a β-1,4-linked d-glucose dimer), are key members of their enzyme cocktails, playing a major role in the hydrolysis of crystalline cellulose.

Glycoside hydrolase (GH) family 6, a class of enzymes defined by amino-acid sequence similarities in the CAZy database (Carbohydrate-Active enZymes; http://www.cazy.org), includes cellobiohydrolases and endoglucanases (EC 3.2.1.4). Structural studies have revealed that cellobio­hydrolases of this family have tunnel-like catalytic sites covered by flexible loops, which are not necessarily conserved in endoglucanases (Rouvinen *et al.*, 1990 ▸; Spezio *et al.*, 1993 ▸). This family is also known for its unusual ‘Grotthus-type’ inverting mechanism: the enzymes appear to lack a general base residue that would activate a catalytic water molecule for direct nucleophilic attack; instead, observations of ordered water molecules near the active site led to the proposal that an additional water molecule is involved in hydrolysis, serving to bridge between the catalytic water molecule and the proton-accepting residue (Koivula *et al.*, 2002 ▸).

The GH family 6 cellobiohydrolase from the basidiomycete *P. chrysosporium* (*Pc*Cel6A) consists of a catalytic domain (CD) and an N-terminal carbohydrate-binding module (CBM) connected by a linker region (Tempelaars *et al.*, 1994 ▸); this modular structure is typical of fungal GH family 6 cellobiohydrolases (Mertz *et al.*, 2005 ▸). In a previous study, we cloned and recombinantly expressed *Pc*Cel6A and found that the hydrolysis rate of *Pc*Cel6A was greatly accelerated by a polymorphic change of crystalline cellulose (Igarashi *et al.*, 2012 ▸). *Pc*Cel6A has been the target of protein engineering aimed at improving its thermostability (Heinzelman *et al.*, 2009 ▸; Ito *et al.*, 2013 ▸), and we have also recently applied random mutagenesis to this enzyme (Tachioka *et al.*, 2016 ▸). Here, we report the structure of the CD of *Pc*Cel6A in its apo form and in complex with the ligand cellobiose.

## 2. Materials and methods   

### 2.1. Expression and purification   

Cloning of the *cel6A* gene from *P. chrysosporium* and the construction of the pPICZα/*cel6A* vector have been described in a previous report (Igarashi *et al.*, 2012 ▸). Deletion of the N-terminal CBM-linker region (residues 1–81) was performed by a combination of inverse PCR and DpnI treatment. The recombinant protein was produced in *P. pastoris* using a 5 l jar fermenter, as reported previously (Igarashi *et al.*, 2012), and was then ultrafiltered and concentrated using a Kvick Lab Cassette 100 kDa and 5 kDa (GE Healthcare, USA). The protein solution in 20 m*M* sodium acetate buffer pH 5.0 containing 1 *M* ammonium sulfate was applied onto a Phenyl Toyopearl 650S column (Tosoh Corporation, Japan) equilibrated with the same buffer. The protein was eluted with a reverse gradient to 20 m*M* sodium acetate buffer pH 5.0 and was then analysed by SDS–PAGE. Doublet bands were seen at approximately 37 kDa, which might reflect a difference in glycosylation. Therefore, the fractions with lower molecular weight were collected. These fractions were equilibrated against 20 m*M* Tris–HCl buffer pH 8.0 and applied onto a DEAE Toyopearl 650S column (Tosoh Corporation) equilibrated with the same buffer. The protein was eluted from the column with a linear gradient from 0 to 20 m*M* NaCl. The purified protein was dialyzed into 5 m*M* Tris–HCl buffer pH 7.5 containing 100 m*M* NaCl.

### 2.2. Crystallization, data collection and structure solution   

Crystallization was performed by the sitting-drop vapour-diffusion method. The drops were formed by mixing 1 µl 20 mg ml^−1^ protein solution with the same volume of a reservoir solution composed of 20%(*w*/*v*) polyethylene glycol 3350, 200 m*M* calcium acetate, 50 m*M* acetate buffer pH 5.0, 10%(*w*/*v*) 2-methyl-2,4-pentanediol. To introduce the ligand, *p*-nitrophenyl β-d-cellotrioside (*p*NPG3) powder was dissolved in mother liquor and a crystal was incubated in this solution for 10 h prior to data collection. X-ray diffraction data sets were collected using synchrotron radiation on beamlines BL5A and BL17A of the Photon Factory, High Energy Accelerator Research Organization (KEK), Tsukuba, Japan. The data sets were processed and scaled using the *HKL*-2000 suite (Otwinowski & Minor, 1997 ▸). The sequence of *Pc*Cel6A was submitted to the *Phyre*2 web server (Kelley & Sternberg, 2009 ▸; http://www.sbg.bio.ic.ac.uk/phyre2) to obtain a suitable search model for molecular replacement with *MOLREP*, an auto-MR function in the *CCP*4 suite (Winn *et al.*, 2011 ▸). Manual model rebuilding and refinement were performed using *Coot* (Emsley *et al.*, 2010 ▸) and *PHENIX* (Adams *et al.*, 2010 ▸). Data-collection and refinement statistics are shown in Table 1 ▸. Most of the molecular-graphics images were prepared using *PyMOL* (v.1.7; Schrödinger). The relative *B* factor was calculated by dividing the average *B* factor of each residue by that of the whole protein: 11.6 Å^2^ for the apo structure and 20.0 Å^2^ for the *Pc*Cel6A–cellobiose structure. The apo and cellobiose-bound structures were superposed using the *phenix.superpose_maps* tool, and the r.m.s.d. values between all C^α^-atom pairs and the relative *B*-factor values were visualized using *UCSF Chimera* (http://www.cgl.ucsf.edu/chimera; Pettersen *et al.*, 2004 ▸). Hydrophobicity was visualized using the *color_h* script in *PyMOL*.

## 3. Results and discussion   

### 3.1. Overall structure of the *Pc*Cel6A catalytic domain   

X-ray data sets were collected from a *Pc*Cel6A crystal and a *p*NPG3-soaked crystal to 1.2 and 2.1 Å resolution, respectively. Both crystals belonged to space group *P*2_1_2_1_2_1_ with one molecule in the asymmetric unit, and the structures were refined to *R*_work_ and *R*_free_ values of 13.8 and 15.9% and of 17.7 and 23.5%, respectively.

**Figure 1:**
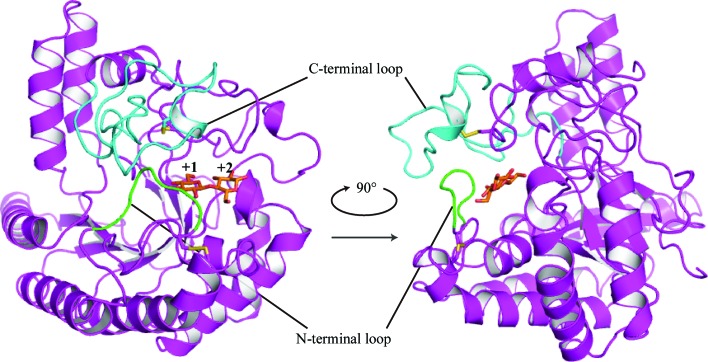
Overall structure of *Pc*Cel6A with cellobiose bound at subsites +1 and +2. The N-terminal and C-terminal loops covering the catalytic centre are coloured green and cyan, respectively.

The CD of *Pc*Cel6A consists of a distorted seven-stranded β/α_8_-barrel, like the CDs of other fungal GH family 6 cellobiohydrolases (Fig. 1 ▸). The final structures included residues 82–439. The catalytic site is located in a tunnel enclosed by a pair of loops (residues 174–178 and 390–425), designated here as the N-terminal and C-terminal loops (shown in green and blue, respectively, in Fig. 1 ▸). A structure-similarity search of the Protein Data Bank (PDB) using the *DALI* server (Holm & Rosenström, 2010 ▸) revealed highly homologous structures among GH family 6 enzymes. The overall structure and ligand-bound structure of *Pc*Cel6A showed the greatest similarity to Cel6A from *Coprinopsis cinerea* (*Cc*Cel6A; Tamura *et al.*, 2012 ▸; PDB entry 3vog), with an r.m.s.d. of 0.7 Å for 357 C^α^ atoms and a *Z*-score of 60.8, and to *Cc*Cel6A–*p*NPG3 (PDB entry 3voi), with an r.m.s.d. of 0.9 Å for 357 C^α^ atoms and a *Z*-score of 61.3, respectively. The sequence similarity between *Pc*Cel6A and *Cc*Cel6A was 65%. The catalytically important residues and two conserved disulfide bridges (Cys171–Cys230 and Cys361–Cys408) are shown in Supplementary Fig. S1, together with sequence alignments of *Pc*Cel6A, *Cc*Cel6A, Cel6A from *Trichoderma reesei* (*Tr*Cel6A) and Cel6A from *Humicola insolens* (*Hi*Cel6A). The CD of *Pc*Cel6A has one potential *N*-glycosylation site at Asn398 according to the *NetNglyc* 1.0 Server (http://www.cbs.dtu.dk/services/NetNGlyc/), but no electron density owing to sugars was visible in the structures, indicating that the proteins used to prepare the crystals were nonglycosylated.

### 3.2. Ligand binding at the +1/+2 sites and conformational change of loops   

**Figure 2:**
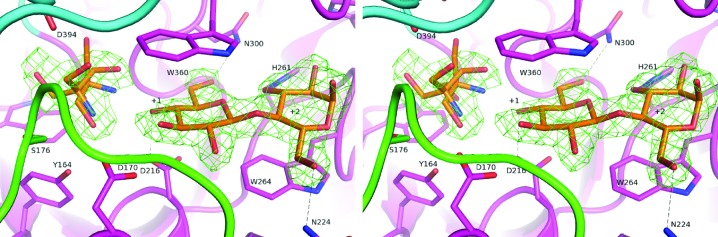
Close-up view of subsites +1 and +2 of *Pc*Cel6A with cellobiose as a ligand. The |*F*_o_| − |*F*_c_| map was calculated without ligand atoms and contoured at the 3σ level. The Tris molecule at subsite −1 was modelled in a double conformation.

The ligand-bound structure obtained from the *p*NPG3-soaked crystal contained one α-cellobiose molecule, which occupies subsites +1 and +2 in the substrate-binding cleft (Fig. 2 ▸). The observed α-cellobiose is considered to be a product of the hydrolysis of *p*NPG3 under the crystallization conditions at pH 5. This is plausible because the GH family 6 enzymes perform hydrolysis of β-1,4-glycosidic bonds with inversion of anomeric configuration, and are known to cleave chromophoric cello-oligosaccharides to produce cellobiose units (Claeyssens *et al.*, 1989 ▸). The cleavage pattern of *p*NPG3 in this structure is different from the *Cc*Cel6A structure, in which nonhydrolyzed *p*NPG3 bound at subsites +1 to +4 (PDB entry 3voi; Tamura *et al.*, 2012 ▸), and from the *Cc*Cel6C structure, in which two *p*NPG2 molecules bind at subsites −3 to −1 and +1 to +3 (PDB entry 3abx; Liu *et al.*, 2010 ▸).

As in other GH family 6 enzymes, the −1 subsite is often occupied by molecules other than waters and sugars, such as cations and low-molecular-weight compounds (Supplementary Table S1), and an unmodelled electron-density blob was similarly observed at the −1 site in the present liganded structure. An octahedrally coordinated Mg^2+^ ion found at subsite −1 of the *Cc*Cel6C–*p*NPG3 structure (PDB entry 3voi), where the ligands only occupied plus-numbered subsites as in this *Pc*Cel6A–cellobiose complex, led us to initially place a hydrated Ca^2+^ ion into the density blob. However, the *B* factor of the hydrated Ca^2+^ ion (48.8 Å^2^) was quite high compared with those of cellobiose (18.5–26.8 Å^2^) and the water molecules around the subsite (approximately 15–30 Å^2^). The automated ligand-identification tool in *PHENIX* (Terwilliger *et al.*, 2006 ▸) predicted Tris as the most favourable of the molecules in the crystallization condition, and the *B* factors for a single Tris molecule were in a reasonable range (29.1–33.4 Å^2^). The second conformation of the Tris molecule was placed manually into the residual electron density of this model, and a molecule with multiple conformations appears to be the most probable explanation, with *B* factors of 18.3–24.8 Å^2^. The Tris molecule made hydrogen bonds to O4 of glucose at subsite +1, the OD1 atom of Tyr164, the NZ atom of Lys388, the carbonyl O atom and the OD1 atom of Asp394 in addition to several water molecules. No water molecule corresponding to the catalytic water was found in the structure because of the binding of the putative Tris molecule.

The tunnel-enclosing N- and C-terminal loops of GH family 6 enzymes have been observed in several conformations in previous crystallographic studies (reviewed in Payne *et al.*, 2015 ▸). Two major conformations of the loops are known, an ‘open’ conformation that makes the subsites more accessible and a ‘closed’ conformation for arrangement of the catalytic site residues into the catalytically competent configuration. In the cellobiose-bound structure, the loops adopted the closed conformation, in contrast to the open conformation observed in the apo structure. As summarized in Supplementary Table S1, the loops may adopt either conformation in enzymes for which the structures are known. The occupation of +1 and +2 sites by sugar moieties results in the closure of the loops, with the exception that proton-accepting residues are ‘off’ conformation and interact with serines on the loop, as in *Hi*Cel6A structures where sugars bind in upside-down configurations (PDB entries 1oc5, 1oc7 and 1ocj; Varrot *et al.*, 2003 ▸).

### 3.3. Noncatalytic but notable residues of *Pc*Cel6A   

**Figure 3:**
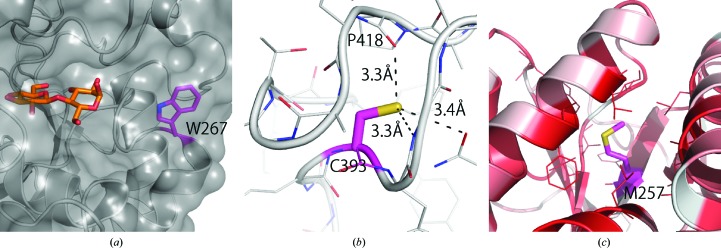
Enlarged views of Trp267 (*a*), Cys393 (*b*) and Met257 (*c*). Each residue is coloured magenta. The red–white colour in (*c*) represents the hydrophobicity of the residues.

Mutagenesis work performed on *Pc*Cel6A revealed several properties of noncatalytic residues of this enzyme (see Heinzelman *et al.*, 2009 ▸; Ito *et al.*, 2013 ▸; Tachioka *et al.*, 2016 ▸). In our recent work, the substitution mutation W267C was found to be a critical mutation for the degradation of crystalline cellulose III_I_ but not of amorphous phosphoric acid-swollen cellulose (Tachioka *et al.*, 2016 ▸), which was a similar finding to the work on *Tr*Cel6A (Koivula *et al.*, 1998 ▸). Trp267 is located at the entrance to the catalytic site at subsite +4 and probably plays a specialized role in the recognition and recruitment of a single cellulose chain from the crystalline surface (Fig. 3 ▸ *a*). Heinzelman and coworkers successfully engineered *Pc*Cel6A to improve its thermostability by a remarkable 10°C by a single mutation of Cys393 to serine (Heinzelman *et al.*, 2009 ▸). This free cysteine makes a weaker hydrogen bond to the carbonyl of Pro418, with a distance of about 3.3 Å (Fig. 3 ▸ *b*), than the serine residues that are found in other homologous fungal structures which originally have a serine instead of cysteine (2.6–2.7 Å in *Chaetomium thermophilum* Cel6A and *Cc*Cel6C), which is consistent with their discussion. Ito and coworkers also improved the thermostability of *Pc*Cel6A by 16 cumulative mutations (Ito *et al.*, 2013 ▸). The largest stabilization of 1.2°C was achieved by a mutation of Met257 to isoleucine, and the authors deduced from modelling *Pc*Cel6A that an exchange to more hydrophobic residues stabilizes the structure in its buried protein environment. Our results reveal that Met257 is surrounded by hydrophobic side chains of α-helix and supports their findings (Fig. 3 ▸ *c*).

### 3.4. Loop flexibility   

**Figure 4:**
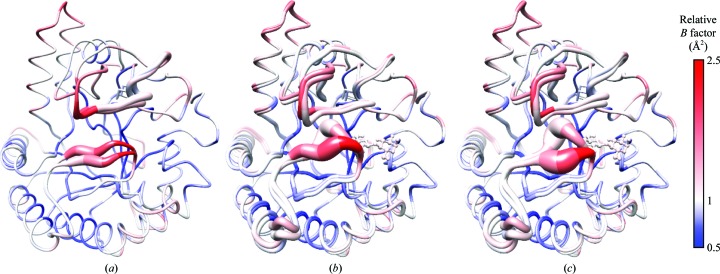
Superposition of the apo and cellobiose-bound structures of *Pc*Cel6A. (*a*) The apo structure was observed in multiple conformations: the ‘relatively open’ and ‘most open’ conformations are shown. (*b*) Superposition of the relatively open apo structure and the cellobiose-bound structure. (*c*) Superposition of the most open apo structure and the cellobiose-bound structure. The width of the ribbon in each figure represents the r.m.s.d. value between the superposed conformations. The relative *B* factors were calculated for each apo and cellobiose-bound structure.

The flexibility and mobility of the loops are important for the catalytic activity. As noted above in §[Sec sec3.2]3.2, the loops of *Pc*Cel6A remained open in the absence of bound ligand, but occupation of the substrate-binding sites induced conformational change of the loops, resulting in a narrowing of the active-site cleft. To further examine the situation, the relative *B* factors of residues and r.m.s.d. values among the observed conformations of *Pc*Cel6A were visualized by means of a blue–red colour scale and worm representations, respectively (Fig. 4 ▸). The residues in the loops have consistently high *B* factors compared with other regions of the protein. As shown in Fig. 4 ▸(*a*), the N- and C-terminal loops of the *Pc*Cel6A apo structure were both modelled as double conformations with similar occupancies ranging from 0.46 to 0.54. In Figs. 4 ▸(*b*) and 4 ▸(*c*), the relatively open and most open apo structures are compared with the cellobiose-bound structure, respectively. The most dynamic conformational change occurred in the N-terminal loop concomitantly with ligand binding, as shown by a ribbon representation, the thickness of which is in proportion to the r.m.s.d values between two conformations. In the C-terminal loop region, on the other hand, the r.m.s.d. values between the apo and cellobiose-bound structures were similar to that between the two conformations of the apo structure. Therefore, both loops showed clear flexibility, but their mobility and response to ligand binding were different.

Although the role of each loop in GH family 6 cellobiohydrolases is not fully understood, it has been suggested that a conformational change of the loops would occur during the catalytic cycle (see Payne *et al.*, 2015 ▸). In this study, we determined both the open and closed structures of the CD of *Pc*Cel6A. Careful analysis of the flexibility and mobility of the N-terminal and C-terminal tunnel-enclosing loops revealed differences in their characteristics, and we speculate that the loops are involved in both the chemical hydrolysis reaction of a single polysaccharide chain and the physical interaction with the crystalline surface of cellulose. The present structural information should afford insight into the structure–function relationship and provide a basis for more detailed mechanistic studies of this industrially and scientifically important enzyme.

## Supplementary Material

PDB reference: *Pc*Cel6A, 5xcy

PDB reference: complex with cellobiose, 5xcz

Supplementary Figure S1 and Table S1.. DOI: 10.1107/S2053230X17008093/nw5054sup1.pdf

## Figures and Tables

**Table 1:** X-ray data-collection and refinement statistics Values in parentheses are for the highest resolution shell.

	*Pc*Cel6A	*Pc*Cel6A–cellobiose
Data collection		
Beamline	BL5A	BL17A
Wavelength (Å)	1.00000	0.98000
Space group	*P*2_1_2_1_2_1_	*P*2_1_2_1_2_1_
Unit-cell parameters		
*a* (Å)	54.7	54.5
*b* (Å)	67.2	67.0
*c* (Å)	89.1	85.1
Resolution (Å)	50.00–1.20 (1.22–1.20)	50.00–2.10 (2.14–2.10)
Total reflections	1014578	129843
Unique reflections	103410	19086
Completeness (%)	99.9 (99.2)	99.5 (99.0)
Multiplicity (%)	3.1 (2.8)	6.8 (6.8)
Average *I*/σ(*I*)	43.7 (5.9)	15.5 (3.1)
*R*_merge_ (%)	5.5 (28.8)	7.2 (48.4)
Mosaicity range (°)	0.26–0.32	0.79–1.27
Refinement		
Resolution (Å)	28.6–1.20 (1.24–1.20)	45.9–2.1 (2.18–2.10)
*R*_work_ (%)	13.8 (14.9)	17.7 (21.9)
*R*_free_ (%)	15.9 (19.6)	23.5 (27.6)
No. of reflections	103210 (10094)	18676 (1806)
No. of atoms	3515	2950
R.m.s.d. from ideal values		
Bond lengths (Å)	0.005	0.007
Bond angles (°)	0.83	0.85
Ramachandran plot		
Favoured regions (%)	96.9	97.0
Additionally allowed (%)	3.1	3.0
Outliers (%)	0	0
