# Supplementary material for: Crystal structure of a family 6 cellobiohydrolase from the basidiomycete Phanerochaete chrysosporium
Source: Acta Crystallogr F Struct Biol Commun. 2017 Jun 17;73(Pt 7):398–403. doi: 10.1107/S2053230X17008093 (PMC5505244; doi:10.1107/S2053230X17008093)
Supplement: Supplementary file 1 [file f-73-00398-sup1.pdf]

Acta Cryst F

STRUCTURAL BIOLOGY COMMUNICATIONS

Volume 73 (2017)

Supporting information for article:

Crystal structure of family 6 cellobiohydrolase from the 2 basidiomycete *Phanerochaete chrysosporium*

Mikako Tachioka, Akihiko Nakamura, Takuya Ishida, Kiyohiko Igarashi and Masahiro Samejima

Supporting information

Table S1 Summary of known structures in the GH family 6.

Enzyme	WT/mutation	PDB code	Subsite																Loop	Catalytic residue*		Year
			-4		-3		-2		-1		+1		+2		+3		+4			Acid	Base	
Phanerochaete chrysosporium Cel6A	WT	5XCY																	open	on	on&off	this
	WT	5XCZ							TRS			O							closed	on	on	this
Trichoderma reesei Cel6A	WT	3CBH																	open			1990
	WT	1QK2						O		S		O							open	off	off	1999
	WT	1QK0						O	Xylp	O	IOB								open	off	off	1999
	Y169F	1CB2																	open	off	off	1996
	Y169F	1QJW						O		S		O							closed	off	on	1999
	D175A	1HGW																	open	off	-	2002
	D221A	1HGY																	closed	-	on	2002
	D221A	4AU0 A										O		O	ClMUF				closed	-	on	2013
	D221A	4AU0 B										O		O	ClMUF				closed	-	on	2013
	D221A	4AX6						O		O	ClPhUF								closed	-	on	2013
	D221A	4AX7 A,C						O		O		O		O	MUF				closed	-	on	2013
	D221A	4AX7 B,D						O		O	MUF								missing	-	on	2013
Humicola insolens Cel6A	WT	1BVW					GOL												open	on&off	off	1999
	WT	2BVW A										O		O		O			closed	on	on	1999
	WT	2BVW B																	closed	on	on	1999
	WT	1OCB		O		S		OMe		H_2N		O		O		S		OMe	closed	on	on	2003
	D416A	1GZ1						O		S		O							open	off	off	2002
	D405N	1OC6			GOL						GOL		GOL						open	off	off	2003
	D405N	1OC5			GOL		GOL		GOL			O		S		O		OMe	open	off	off	2003
	D405N	1OC7								S		S		S		S		OMe	open	off	off	2003
	D416A	1OCJ				S		S		S		S		OMe					open	off	off	2003
	D416A	1OCN						O	IFM			O	IFM						closed	on	on	2003
Coprinopsis cinerea Cel6A	WT	3VOG							HEPES										open	off	off	2012
	WT	3VOH				O						O		O					closed	on	on	2012
	WT	3VOI							Mg^{2+}			O		O		O	*p*NP		closed	on	on	2012
	D164A	3VOJ																	closed	off	-	2012
Coprinopsis cinerea Cel6C	WT	3A64																	open	off	off	2010
	WT	3ABX				O		O	*p*NP					O		O	*p*NP		open	off	off	2010
	WT	3A9B										O							open	on & off	off	2010
	D102A	3VOF																	closed	off	-	2012
Chaetomium thermophilum Cel6A	WT	4A05				O			Li^+			O		O		O			closed	on	on	2012
Thermobifida fusca Cel6B	WT	4B4H																	open	off	off	2013
	WT	4B4F A						O		O		O		O		O			closed	off	on	2013
	WT	4B4F B						O		O		O							closed	off	on	2013
	D274A	4AVO						O		O		O		O		O			closed	-	on	2013
	D226A/S232A	4AVN										O		O		O			closed	off	-	2013
Humicola insolens Cel6B	WT	1DYS																	open	on	off	2000
Thermobifida fusca Cel6A	WT	1TML																		on	***	1993
	WT	2BOD						O		S		O							missing	on	***	2005
	Y73S	2BOE																	missing	on	***	2005
	Y73S	2BOF						O		O		O							missing	on	***	2005
	Y73S	2BOG						O		S		O		OMe					missing	on	***	2005
Mycobacterium tuberculosis Cel6	WT	1UP0																	closed?	on	on	2005
	WT	1UP3										O		S	**	O	**	OMe	closed?	on	on	2005
	WT	1UOZ						GOL				S		S	**	SH			closed?	on	on	2005
	WT	1UP2						O	IFM			O	IFM						closed?	on	on	2005

Glucopyranose   Glucopyranose (upside down)   Aglycone

* Acid and base represent the catalytic acid and proton-accepting residue in the Grotthuss mechanism, respectively. On and off indicate conformations of the residues; pointing toward and away from the center of the active-site tunnel, respectively.

** Glucopyranoses have no interaction with the protein because of the lack of subsites +3 and +4 in *M. tuberculosis* Cel6.

***The Asp79 in *T. fusca* Cel6A is distantly positioned from catalytic center.

Abbreviations used for aglycone: ClMUF, 6-chloro-4-methyl-umbelliferone; ClPhUF, 6-chloro-4-phenyl-umbelliferone; IFM, 5-hydroxymetyl-3,4-dihydroxypiperidine; IOB, 3-iodo-benzylalcohol; MUF, methyl-umbelliferone; Me, methyl group; *p*NP, *p*-nitrophenol; Xylp, xylopyranose.

Abbreviations used for other molecules: GOL, glycerol; HEPES, 4-(2-hydroxymethyl)-1-piperazine ethanesulfonic acid; TRS, 2-amino-2-hydroxymethyl-propane-1,3-diol.

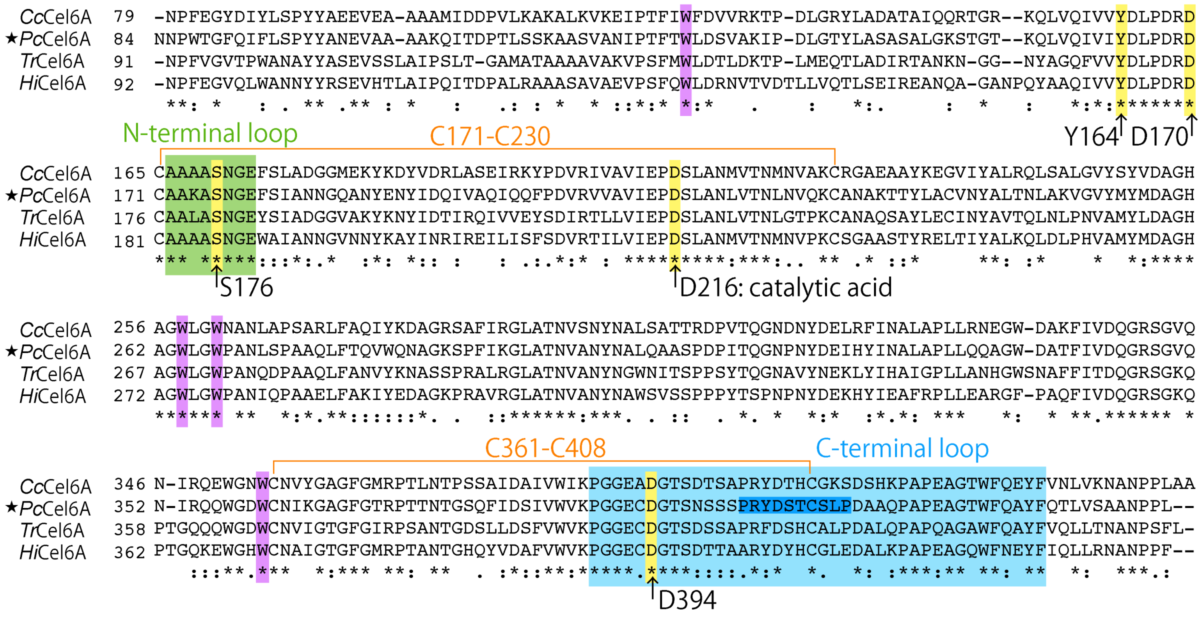

Figure S1 Sequence alignment of the catalytic domains of *P. chrysosporium* Cel6A, *C. cinerea* Cel6A, *T. reesei* Cel6A and *H. insolens* Cel6A. The catalytically important residues are highlighted in yellow, and residue numbers of *Pc*Cel6A are shown with arrows. The N-terminal and C-terminal loops are colored in green and cyan, respectively, and the regions observed in multiple conformations in *Pc*Cel6A are highlighted in darker colors.
